# Hyperuricemia reduces the risk of MCI but not dementia: a cross-sectional study in Liuyang

**DOI:** 10.3389/fneur.2025.1555587

**Published:** 2025-03-17

**Authors:** Yong He, Tieshi Zhu, Erxinxian Bei, Guangpeng Xiang, Danyang Xi, Heng Meng, Yuzhang Bei

**Affiliations:** ^1^Department of Neurology, The First Affiliated Hospital, Jinan University, Guangzhou, China; ^2^Department of Neurology, Liuyang Jili Hospital, Changsha, China; ^3^Department of Neurology, Zhanjiang Central Hospital, Guangdong Medical University, Zhanjiang, China; ^4^Department of Neurology, Haikou Affiliated Hospital of Central South University Xiangya School of Medicine, Haikou, China; ^5^Department of Hematology, Zhanjiang Central Hospital, Guangdong Medical University, Zhanjiang, China; ^6^Department of Anesthesiology, Zhanjiang Central Hospital, Guangdong Medical University, Zhanjiang, China

**Keywords:** hyperuricemia, cognitive impairment, mild cognitive impairment, dementia, cross-sectional study

## Abstract

**Background:**

Cognitive impairments, including mild cognitive impairment (MCI) and dementia, significantly affect patients’ daily functions and quality of life, contributing to a substantial societal and economic burden. The role of uric acid in cognitive function is controversial, with some studies suggesting protective effects, while others indicate increased risk of cognitive decline.

**Methods:**

A total of 1,098 participants with an average age of 69 years were included in the study. Cognitive function was assessed using the Mini-Mental State Examination. Hyperuricemia was defined as blood uric acid concentrations >420 μmol/L. Logistic regression and restricted cubic spline analysis were performed to assess the association between hyperuricemia and cognitive impairment, including MCI and dementia.

**Results:**

Hyperuricemia was associated with a lower risk of cognitive impairment (OR = 0.51, 95% CI = 0.31–0.80) and MCI (OR = 0.39, 95% CI = 0.21–0.69), particularly in individuals younger than 70 years, males, and those without hypertension or diabetes. No significant association was found between hyperuricemia and dementia (OR = 0.94, 95% CI = 0.44–1.89). There is no evidence of a non-linear relationship between hyperuricemia and cognitive impairment.

**Conclusion:**

Hyperuricemia appears to have a protective effect on cognitive function, particularly in reducing the risk of MCI, but not dementia, in specific populations.

## 1 Introduction

Cognitive impairments, including mild cognitive impairment (MCI) and dementia, significantly reduce patients’ ability to work and perform daily activities, leading to an increased societal and economic burden (1–3). Recent studies have identified oxidative stress as a significant factor contributing to cognitive impairments (4, 5).

Uric acid is a naturally occurring antioxidant in the body, and its antioxidative properties have long been recognized (6–9). However, the role of uric acid in cognitive impairment remains controversial, with conflicting findings in the literature (10). Some studies have indicated that elevated uric acid levels are associated with an increased risk of cognitive decline (11, 12), while others have suggested a protective effect of uric acid on cognitive function (13, 14). This discrepancy may stem from the dual nature of uric acid’s effects. On one hand, uric acid acts as a free radical scavenger and alleviates oxidative stress, providing neuroprotective benefits (15–17). On the other hand, when present in excessive amounts, uric acid may exhibit pro-oxidant and pro-inflammatory properties, leading to endothelial cell damage, promoting neuroinflammation, and contributing to neuronal injury (16, 18, 19).

Given this complex and multifaceted role of uric acid, further investigation into its relationship with cognitive impairment is essential. This study aims to analyze the concentrations at which uric acid exerts either beneficial or harmful effects, thereby clarifying the relationship between uric acid and cognitive function, addressing conflicting findings, and ultimately offering guidance for therapeutic strategies to prevent or mitigate cognitive impairment.

## 2 Methods

### 2.1 Population

The study population was recruited from the Liuyang Jili Hospital Physical Examination Center in Liuyang, China, consisting of residents from townships and communities in Liuyang. A total of 1,138 individuals participated, including 524 males and 614 females, with an average age of approximately 69 years. Due to missing data, 36 participants were excluded for incomplete dietary questionnaires, and 4 were excluded for lacking blood test data, resulting in a final sample size of 1,098 individuals for analysis.

### 2.2 Outcome

Cognitive function assessment was conducted at the Liuyang Jili Hospital Physical Examination Center using the Mini-Mental State Examination (MMSE) scale (20). The scoring criteria were as follows: for MCI — illiterate individuals scored between 14 and 17, elementary school graduates scored between 17 and 19, and those with junior high school education or above scored between 20 and 24. For dementia — illiterate individuals scored between 0 and 13, elementary school graduates scored between 0 and 16, and those with junior high school education or above scored between 0 and 19 (21).

### 2.3 Exposure

Blood uric acid concentrations were obtained from measurements conducted at the Liuyang Jili Hospital Physical Examination Center. According to the expert consensus and guidelines on hyperuricemia in China, hyperuricemia was defined as a blood uric acid concentration > 420 μmol/L, corresponding to the “High” group in Table 1.

**Table 1:** Baseline characteristics by uric acid.

	Normal (*n* = 904)	High (*n* = 194)	*p*
Age (year)	69.03 (6.01)	69.46 (6.63)	0.378
Sex			<0.001
Female	534 (59.1)	64 (33.0)	
Male	370 (40.9)	130 (67.0)	
Cognitive status			0.006
Dementia	62 (6.9)	12 (6.2)	
MCI	150 (16.6)	15 (7.7)	
Normal	692 (76.5)	167 (86.1)	
Body mass index	24.17 (3.03)	25.54 (3.67)	<0.001
Physical exercise			0.213
Never	398 (44.0)	94 (48.5)	
Every day	468 (51.8)	89 (45.9)	
Sometime	38 (4.2)	11 (5.6)	
Diet			0.192
Meat-based	1 (0.1)	0 (0.0)	
Balanced	888 (98.2)	187 (96.4)	
Vegetarian	15 (1.7)	7 (3.6)	
Smoke			0.113
Never	709 (78.4)	141 (72.7)	
Former	35 (3.9)	13 (6.7)	
Now	160 (17.7)	40 (20.6)	
Drink			0.130
Never	824 (91.2)	167 (86.1)	
Frequent	10 (1.1)	2 (1.0)	
Every day	29 (3.2)	9 (4.6)	
Sometime	41 (4.5)	16 (8.2)	
Hypertension			<0.001
Yes	422 (46.7)	120 (61.9)	
No	482 (53.3)	74 (38.1)	
Diabetes			0.357
Yes	132 (14.6)	34 (17.5)	
No	772 (85.4)	160 (82.5)	
Hemoglobin (g/L)	134.93 (13.31)	136.60 (15.45)	0.124
NLR	2.04 (0.89)	2.23 (0.99)	0.01
Albumin (g/L)	41.36 (2.92)	41.11 (2.96)	0.278
Creatinine (μmol/L)	69.19 (38.77)	94.86 (51.60)	<0.001
Blood urea nitrogen (mmol/L)	5.32 (1.43)	6.44 (2.90)	<0.001
Uric acid (μmol/L)	311.95 (59.22)	487.98 (58.63)	<0.001

Data are presented as mean values (SD) or *n* (%). SD: standard deviation; MCI: Mild cognitive impairment; NLR: Neutrophil-lymphocyte ratio.

### 2.4 Other covariates

Age and sex were recorded at the time of the physical examination. Body mass index (BMI) was calculated using the height and weight measurements taken at the Physical Examination Center. Information on the frequency of physical exercise, dietary habits, smoke, drink, hypertension, and diabetes was collected through interviews conducted by medical examiners during the physical examination. Hemoglobin, albumin, creatinine, and blood urea nitrogen levels were obtained from blood tests performed at the Medical Examination Center. The neutrophil-to-lymphocyte ratio (NLR) was calculated based on routine blood test results provided by the Medical Examination Center (22, 23).

### 2.5 Statistics

Data analysis was performed using R version 4.4.1. Continuous variables were compared using either Student’s t-test or the nonparametric Mann–Whitney U test, while categorical variables were analyzed using the chi-square test or Fisher’s exact test. Logistic regression models were employed to assess the association between hyperuricemia and the risk of cognitive impairment, with the results reported as odds ratios (ORs) with 95% confidence intervals (CI) and *p*-values. Restricted Cubic Spline (RCS) analysis was conducted to evaluate potential nonlinear relationships.

The associations between hyperuricemia and cognitive impairment, including MCI and dementia, as well as the relationships with MCI and dementia, were analyzed separately. We constructed four models, using the normal uric acid group as the reference. Model 1 was unadjusted. Model 2 was adjusted for age, sex, and BMI. Model 3 was further adjusted for physical exercise, diet, smoke, drink, hypertension, and diabetes. Model 4 additionally adjusted for hemoglobin, NLR, albumin, creatinine, and blood urea nitrogen. In the subgroup analyses, adjustments were consistent with Model 4, excluding subgroup-specific variables. The RCS analyses also incorporated the same covariates as Model 4.

## 3 Results

### 3.1 Baseline characteristics stratified by uric acid levels

Table 1 summarizes baseline characteristics by uric acid levels. The high uric acid group (*n* = 194, mean uric acid = 487.98 μmol/L) had a significantly higher proportion of males (67.0% vs. 40.9%, *p* < 0.001) and higher BMI (25.54 vs. 24.17, *p* < 0.001) compared to the normal group (*n* = 904, mean uric acid = 311.95 μmol/L). Age did not differ significantly between groups (69.46 vs. 69.03 years, *p* = 0.378). The prevalence of mild cognitive impairment and dementia was lower in the high uric acid group (MCI: 7.7% vs. 16.6%; dementia: 6.2% vs. 6.9%, *p* = 0.006). No significant differences were found in physical activity, dietary habits, smoking, alcohol consumption, or diabetes prevalence. Hypertension was more common in the high uric acid group (61.9% vs. 46.7%, *p* < 0.001). Hemoglobin and albumin levels were similar between groups, while NLR (2.23 vs. 2.04, *p* = 0.01), serum creatinine (94.86 vs. 69.19 μmol/L, *p* < 0.001), and blood urea nitrogen (6.44 vs. 5.32 mmol/L, *p* < 0.001) were significantly higher in the high uric acid group.

### 3.2 Hyperuricemia reduces the risk of cognitive impairment

**Figure 1:**
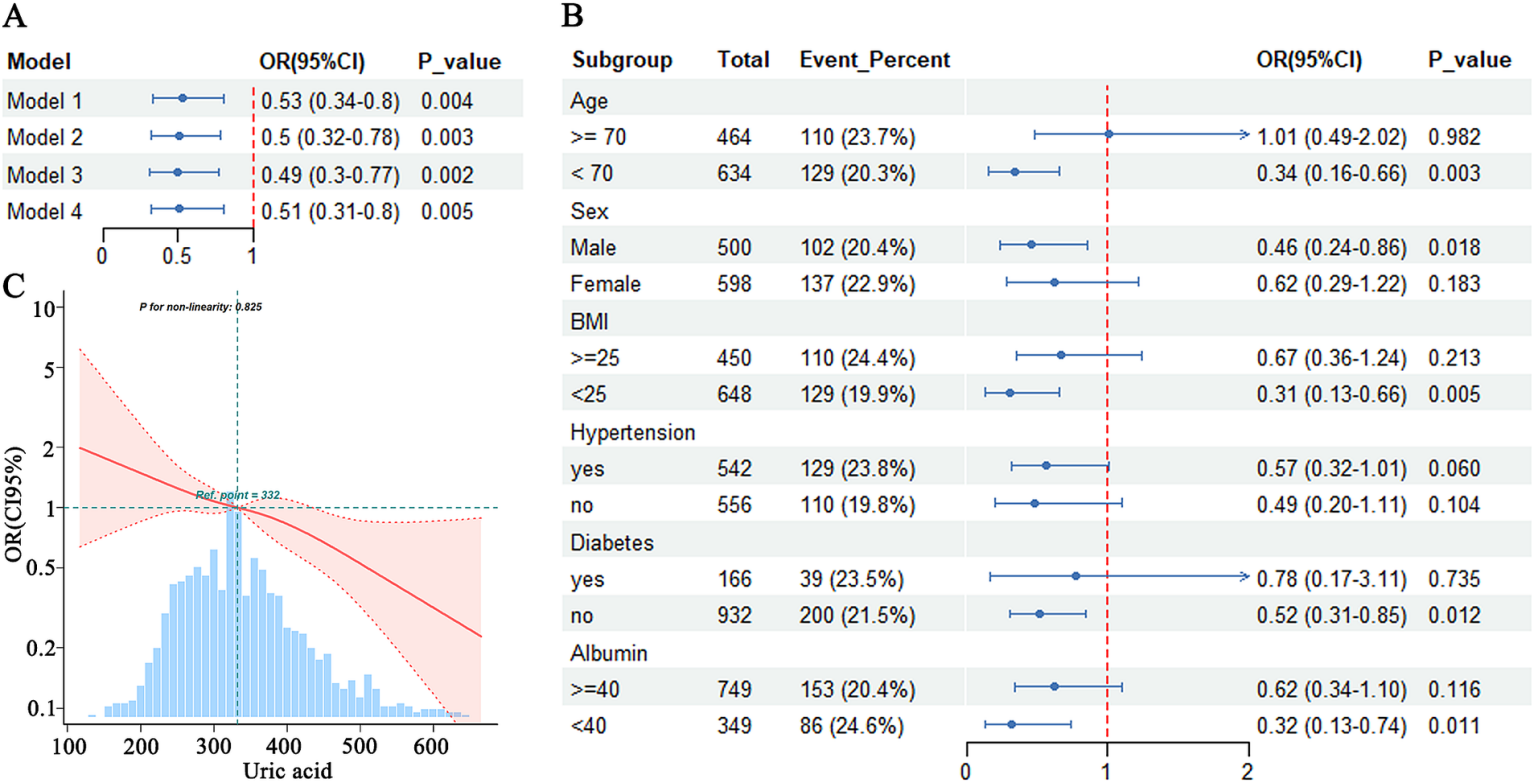
Association between hyperuricemia and cognitive impairment. **(A)** Logistic regression analysis of hyperuricemia and cognitive dysfunction. **(B)** Subgroup analysis of hyperuricemia and cognitive impairment. **(C)** RCS analysis of uric acid and cognitive impairment.

When both MCI and dementia were analyzed together as cognitive impairment, hyperuricemia was found to significantly reduce the risk of cognitive impairment, even after adjusting for multiple covariates in Model 4 (OR = 0.51, 95% CI = 0.31–0.80) (Figure 1A). Subgroup analyses indicated that the association between hyperuricemia and cognitive impairment varied significantly across different characteristics. A significant negative association between hyperuricemia and cognitive impairment was observed in individuals aged <70 years (OR = 0.34, 95% CI = 0.16–0.66, *p* = 0.003), males (OR = 0.46, 95% CI = 0.24–0.86, *p* = 0.018), those with a BMI <25 (OR = 0.31, 95% CI = 0.13–0.66, *p* = 0.005), those without hypertension (OR = 0.49, 95% CI = 0.20–1.11, *p* = 0.094), those without diabetes (OR = 0.52, 95% CI = 0.31–0.85, *p* = 0.012), and those with albumin levels <40 g/L (OR = 0.32, 95% CI = 0.13–0.74, *p* = 0.011), suggesting that hyperuricemia may be protective in these specific populations (Figure 1B). However, the association between hyperuricemia and cognitive impairment did not reach statistical significance in individuals aged ≥70 years, females, those with a BMI ≥25, those with hypertension, and those with diabetes, suggesting that the protective effect of hyperuricemia may not be significant in these populations (Figure 1B).

RCS analysis demonstrates that there was no evidence of a nonlinear relationship between uric acid concentration and the risk of cognitive impairment (p-nonlinearity = 0.825). The risk of cognitive impairment was progressively reduced with increasing uric acid concentrations, without a clear inflection point (Figure 1C).

### 3.3 Hyperuricemia reduces the risk of MCI

**Figure 2:**
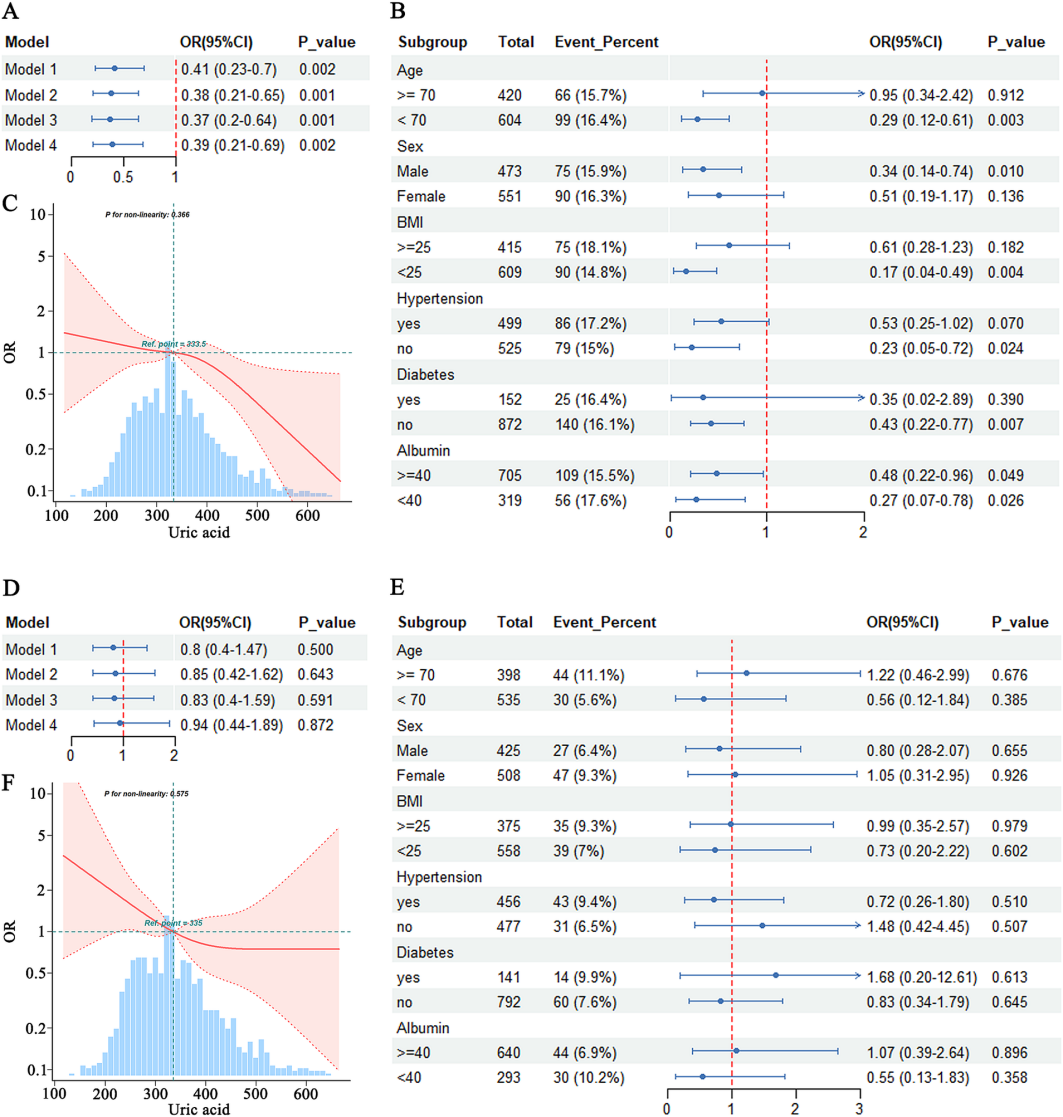
Association between hyperuricemia and MCI **(A–C)** and Dementia **(D–F)**. **(A)** Logistic regression analysis of hyperuricemia and MCI. **(B)** Subgroup analysis of hyperuricemia and MCI. **(C)** RCS analysis of uric acid and MCI. **(D)** Logistic regression analysis of hyperuricemia and dementia. **(E)** Subgroup analysis of hyperuricemia and dementia. **(F)** RCS analysis of uric acid and dementia.

The logistic regression analysis indicated that hyperuricemia significantly reduced the risk of MCI compared to the normal group, even after adjustment for multiple covariates in Model 4 (OR = 0.39, 95% CI = 0.21–0.69) (Figure 2A). Subgroup analyses showed that the effect of hyperuricemia on MCI varied across different characteristics. Hyperuricemia was associated with a reduced risk of MCI in individuals aged <70 years (OR = 0.29, 95% CI = 0.12–0.61, *p* = 0.003), males (OR = 0.34, 95% CI = 0.14–0.74, *p* = 0.010), those with a BMI <25 (OR = 0.17, 95% CI = 0.04–0.49, *p* = 0.004), those without hypertension (OR = 0.23, 95% CI = 0.05–0.72, *p* = 0.024), those without diabetes mellitus (OR = 0.43, 95% CI = 0.22–0.77, *p* = 0.007), and those with albumin levels <40 g/L (OR = 0.27, 95% CI = 0.07–0.78, *p* = 0.026) (Figure 2B). A significant negative association was also observed between hyperuricemia and MCI in individuals with albumin levels ≥40 g/L (OR = 0.48, 95% CI = 0.22–0.96, *p* = 0.049). However, no significant association was found in individuals aged ≥70 years (OR = 0.95, 95% CI = 0.34–2.42, *p* = 0.912), females (OR = 0.51, 95% CI = 0.19–1.17, *p* = 0.136), those with a BMI ≥25 (OR = 0.61, 95% CI = 0.28–1.23, *p* = 0.182), those with hypertension (OR = 0.53, 95% CI = 0.25–1.02, *p* = 0.070), and those with diabetes mellitus (OR = 0.35, 95% CI = 0.02–2.89, *p* = 0.390) (Figure 2B).

RCS analysis indicated that there was no evidence of a nonlinear relationship between uric acid levels and MCI (p-nonlinearity = 0.366). Overall, higher uric acid levels were associated with a lower risk of MCI (Figure 2C).

### 3.4 Hyperuricemia not associated with dementia risk

The analysis results indicated that hyperuricemia was not associated with the risk of dementia in either Model 1 (OR = 0.80, 95% CI = 0.40–1.47) or Model 4 (OR = 0.94, 95% CI = 0.44–1.89) (Figure 2D). No significant associations were found in any of the subgroups, including age (≥70 years: OR = 1.22, 95% CI = 0.46–2.99, *p* = 0.676; <70 years: OR = 0.56, 95% CI = 0.12–1.84, *p* = 0.385), sex (male: OR = 0.80, 95% CI = 0.28–2.07, *p* = 0.655; female: OR = 1.05, 95% CI = 0.31–2.95, *p* = 0.926), BMI (≥25: OR = 0.99, 95% CI = 0.35–2.57, *p* = 0.979; <25: OR = 0.73, 95% CI = 0.28–1.70, *p* = 0.463), hypertension status (with hypertension: OR = 0.72, 95% CI = 0.26–1.80, *p* = 0.510; without hypertension: OR = 1.48, 95% CI = 0.42–4.35, *p* = 0.507), diabetes status (with diabetes: OR = 1.68, 95% CI = 0.20–12.61, *p* = 0.613; without diabetes: OR = 0.83, 95% CI = 0.34–1.97, *p* = 0.645), and albumin levels (≥40 g/L: OR = 1.07, 95% CI = 0.39–2.64, *p* = 0.896; <40 g/L: OR = 0.55, 95% CI = 0.13–1.83, *p* = 0.329) (Figure 2E).

RCS analysis indicated that there was no evidence of a nonlinear relationship between uric acid levels and the risk of dementia (p-nonlinearity = 0.575), although the graphical representation suggested a trend of decreasing dementia risk with increasing uric acid levels (Figure 2F).

## 4 Discussion

In this study, a retrospective analysis of data from the Liuyang Jili Hospital Physical Examination Center indicated that hyperuricemia significantly reduced the risk of cognitive dysfunction, including both MCI and dementia. Further analysis revealed that hyperuricemia was significantly associated with a reduced risk of MCI, particularly among individuals younger than 70, without hypertension or diabetes, and males, whereas no significant association was found with the risk of dementia. In the RCS analysis, uric acid concentration was inversely associated with the risk of cognitive impairment, without evidence of a nonlinear relationship.

As previously mentioned, uric acid is a natural antioxidant in the body that counteracts oxidative stress, though in certain situations it can also exert pro-oxidant and pro-inflammatory effects (7, 16, 24). Currently, uric acid is recognized as an independent risk factor for cardiovascular disease (25, 26). This is supported by our finding that the prevalence of hypertension was higher in the high uric acid group compared to the normal uric acid group. However, the relationship between uric acid and cognitive function remains controversial, with some studies presenting conflicting results.

A prospective study conducted in the Ommoord district of Rotterdam, the Netherlands, included 4,618 participants with a mean age of 69 years and a mean follow-up period of 11.1 years. The study found that uric acid levels were negatively associated with the risk of developing dementia (HR = 0.89 for each standard deviation increase in uric acid, 95% CI = 0.80–0.99) (27). Another study from Taiwan analyzed 28,769 gout patients over 50 years old and 114,742 controls (mean age 63.5 years). After six years of follow-up, the study concluded that gout patients had a lower risk of developing non-vascular dementia (HR: 0.77; 95% CI: 0.72–0.83; *p* < 0.001) and vascular dementia (HR: 0.76; 95% CI: 0.65–0.88; *p* < 0.001) (28). The results of these studies are both consistent and inconsistent with the present study. Our study found that elevated uric acid primarily reduced the risk of mild cognitive impairment, though no significant reduction in the risk of dementia was observed. However, the RCS analysis indicated that higher uric acid levels were associated with a reduced risk of cognitive impairment. In contrast, another study from the Atherosclerosis Risk in Communities cohort, which followed 11,169 participants without dementia or cardiovascular disease for a median of 24.1 years, reported that higher uric acid levels were associated with accelerated cognitive decline (25-year global Z-score difference of −0.149; 95% CI: −0.246, −0.052), but not necessarily a higher risk of developing dementia (HR: 1.03; 95% CI: 0.88–1.21) (11). The controversy arises from heterogeneity in the studied populations, including differences in age distribution, comorbid conditions such as metabolic syndrome and hypertension, and the intricate interplay among oxidative stress, inflammation, and vascular integrity.

Based on our findings, we are inclined to support the idea that elevated uric acid levels may serve as a protective factor for cognitive function, consistent with several meta-analyses. One meta-analysis, which included 46 studies with a total of 16,688 participants on all-cause dementia and 22 studies on attention deficits, concluded that uric acid exerts a protective effect on cognitive function (29). Another meta-analysis by Du et al., which included 21 case–control studies and 3 cohort studies with a total of 10,953 subjects, found that higher uric acid levels were significantly associated with a reduced risk of AD (RR = 0.66, 95% CI: 0.52–0.85, *p* = 0.001) (30).

However, our study also found that elevated uric acid levels did not seem to reduce the risk of dementia, which may be related to the vascular risk associated with uric acid. In our analysis, the prevalence of hypertension was higher in the high uric acid group compared to the normal uric acid group, potentially increasing the risk of vascular dementia, especially among older adults. A large community-based cohort study of healthy older adults, which included 1,598 individuals and followed them for a median of 10.1 years, found that hyperuricemia was associated with a higher risk of vascular or mixed dementia (HR = 3.66, 95% CI: 1.29–10.41, *p* = 0.015), as well as with extensive cerebral white matter lesions (31). High serum uric acid levels may increase the risk of vascular dementia but not AD (31, 32). This can be understood considering that hypertension significantly increases the likelihood of cerebral white matter lesions, while controlling hypertension can slow their progression (33–35).

In the subgroup analyses conducted in this study, the association between elevated uric acid levels and mild cognitive impairment was not significant in subgroups including individuals aged 70 years or older, females, those with a BMI of 25 or higher, as well as those with hypertension or diabetes. We believe this may be because these individuals have a greater number of risk factors for vascular dementia. Although elevated uric acid may provide some protective effects, these benefits may be offset by other detrimental risk factors. This finding aligns with the study by Tuven et al., which reported that higher uric acid levels were associated with better cognitive function in individuals without cardiovascular risk factors that might otherwise attenuate the potential neuroprotective effects of uric acid (36).

The precise mechanism by which uric acid modulates cognitive function remains inconclusive but is hypothesized to involve oxidative stress, amyloid-*β* (Aβ) expression, and vascular endothelial dysfunction. Animal studies have demonstrated that uric acid can reduce oxidative stress products in dopaminergic neurons while enhancing superoxide dismutase activity, thereby mitigating 6-hydroxydopamine-induced neurotoxicity (37). However, other reports suggest that when uric acid levels exceed a certain threshold, it exhibits pro-oxidant properties that may impair cognitive function (38). The pro-oxidant effects of uric acid have been associated with increased expression of inflammatory markers such as C-reactive protein and interleukin-6, vascular endothelial damage, and microvascular dysfunction, potentially compromising cerebral perfusion (39–41). Some studies suggest that cortical ischemia may serve as a key mediator of the relationship between uric acid and cognitive function (42). A prospective population-based study reported that higher uric acid levels were associated with reduced cerebrospinal fluid Aβ levels and improved MMSE scores. However, in contrast, cellular studies have shown that uric acid promotes Aβ expression and exerts neurotoxic effects (43, 44). These findings underscore the bidirectional nature of the relationship between uric acid and cognitive impairment, suggesting that its effects may depend on physiological context and disease state.

Another question arises: if elevated uric acid reduces the risk of cognitive impairment, is medication still necessary? Unfortunately, this study does not provide a definitive answer to this question. However, medications such as benzbromarone and febuxostat could be considered, as previous studies have shown that both drugs reduce the risk of dementia in patients with gout (45, 46).

This study has several limitations. First, only a single measurement of uric acid was taken, which limits the ability to dynamically observe long-term uric acid levels. Second, the assessment of cognitive function relied solely on the MMSE scale, which is not ideal given the complexity of cognition—using a single scale may not provide a comprehensive assessment. Additionally, as a cross-sectional study, this research can only demonstrate correlation rather than causation; more prospective studies are needed to establish causal relationships. Further research is also needed to explore the impact of uric acid-lowering drugs on cognitive function.

Despite its limitations, this study remains significant. By demonstrating that elevated uric acid reduces the risk of MCI rather than the risk of dementia, and by conducting subgroup analyses that highlight the potential protective effect of elevated uric acid on cognitive function in specific populations, this study contributes to the understanding of the complex relationship between uric acid and cognitive function. This is particularly valuable given the scarcity of studies focused on grassroots communities in China.

## 5 Conclusion

In this study, we analyzed data from the Liuyang Jili Hospital Physical Examination Center and concluded, through logistic regression and RCS analyses, that elevated uric acid levels reduce the risk of MCI but not dementia. This protective effect was particularly evident among individuals younger than 70 years, males, those with a BMI below 25, and those without diabetes or hypertension.

## Data Availability

The datasets presented in this article are not readily available because the data used in this study were obtained from the Liuyang Jili Hospital Medical Examination Center, and their use was contingent upon obtaining ethical approval from the hospital. Without this approval, the data could not be used, and therefore cannot be made publicly available. The authors do not have permission to share data. Requests to access the datasets should be directed to Yong He, 277475748@qq.com.
